# Metagenome Sequences from Tidal Marsh and Marine Sediment from the Great Bay Estuary of New Hampshire

**DOI:** 10.1128/MRA.00038-20

**Published:** 2020-03-05

**Authors:** Brian M. Moore, Sinéad M. Ní Chadhain, Jarrett L. Miller, Stephen H. Jones, Loren A. Launen

**Affiliations:** a Department of Biology, Keene State College, Keene, New Hampshire, USA; b Department of Biology, University of South Alabama, Mobile, Alabama, USA; c Department of Natural Resources and the Environment, University of New Hampshire, Durham, New Hampshire, USA; Georgia Institute of Technology

## Abstract

Tidal marsh and estuarine marine microbial sediment metagenomes from the Great Bay Estuary of New Hampshire were sequenced and found to be dominated by *Proteobacteria*, *Bacteroidetes*, *Firmicutes*, and *Actinobacteria.* Both types of sediment contained many unclassified bacterial sequences, including the mollusk pathogen Perkinsus marinus, and detectable xenobiotic degradation and nitrogen transformation genes.

## ANNOUNCEMENT

The Great Bay Estuary (GBE) of New Hampshire is a 2,600-km^2^ recessed estuary in the Gulf of Maine experiencing declining eelgrass, oyster, and clam populations and nutrient loading and contamination from a variety of pollutants (1). The sediment microbial communities in the GBE have not yet been well studied, even though others have shown that estuarine microbial communities are important in estuarine health (2–6). Using a metagenomic approach, we characterized two sediment microbial communities from the GBE, an urban brackish fringing marsh (Coco) contaminated with xenobiotics (Coco [7]; 43.197444 N, 70.867556 W) and a marine sediment adjacent to a natural oyster bed off Nannie Island, distant from pollution sources (43.068472 N, 70.863333 W).

At each site, several sediment grab samples were collected from the entire top 25 cm within a 2- by 4-m area and transported on ice to the lab, and all grab samples from each site (surface through to 25-cm depth) were composited immediately as described previously (7). Composite samples were analyzed for total petroleum hydrocarbons (Eastern Analytical, Inc.), which were present at 320 mg/kg (Coco) and <40 mg/kg (nondetectable; NI). Genomic DNA was extracted using the Mo Bio PowerSoil DNA kit. Library preparation (TruSeq) and metagenome shotgun sequencing (100-bp paired ends) of DNA were performed by the Advanced Genome Technology Core, Vermont Cancer Center, University of Vermont, using an Illumina HiSeq 1000 instrument. Reads were analyzed using the Metagenomics Rapid Annotations using Subsystems Technology (MG-RAST; Table 1) server version 4.0.2 using default parameters (8).

**TABLE 1:** Basic sequence statistics and quality information (MG-RAST)

Sample	No. of sequences				
	Initial (raw)	Passed QC^*a*^	rRNA	Known predicted proteins	Unknown predicted proteins
Coco	146,686,138	117,206,079	133,533	23,876,157	84,318,919
NI	405,221,636	329,228,599	456,991	62,960,339	240,245,010

a QC, quality control.

Taxonomic profiling at the domain level showed predominantly bacterial sequences (95.64% and 96.07% for Coco and NI, respectively). The dominant bacterial phyla in both sediments were *Proteobacteria* (54.15% and 64.17%, respectively) and *Bacteroidetes* (9.28% and 10.33%, respectively) for Coco and NI. Following these, the Coco data set contained *Chloroflexi* (5.66%), *Firmicutes* (5.08%), *Actinobacteria* (4.66%), *Verrucomicrobia* (4.43%), *Planctomycetes* (2.65%), and *Acidobacteria* (2.09%), with *Chlorobi*, *Spirochaetes*, and *Nitrospirae* present at <1% each and 0.84% of sequences not classified at the phylum level. The NI data set contained *Firmicutes* (3.90%), *Planctomycetales* (3.37%), *Actinobacteria* (2.83%), and *Chloroflexi* (2.65%), with *Verrucomicrobia*, *Cyanobacteria*, and *Acidobacteria* each amounting to 1 to 2% of the sequences. Sequences matching the archaeal phylum *Euryarchaeota* were abundant in both sediments (3.04% in Coco and 1.26% in NI). The mollusk alveolate pathogen Perkinsus marinus was present at both sites (<0.01%). NI contained 0.97% reads mapping to the diatom phylum *Bacillariophyta*.

Functional analysis mapped 12.4% (Coco) and 10.8% (NI) of the reads to the Kyoto Encyclopedia of Genes and Genomes (KEGG) Orthology database. Of these, 0.8% (both Coco and NI) were assigned to xenobiotic degradation, including nitrotoluene, toluene, chlorocyclohexane, chlorobenzene, atrazine, polycyclic aromatic hydrocarbons, benzoate and aminobenzoate, xylene, ethylbenzene, dioxin, alkanes, chloroalkanes, chloroalkenes, atrazine, dichlorodiphenyltrichloroethane (DDT), caprolactam, and styrene degradation. Nitrogen metabolism-associated reads were found at the same level in both samples (0.6%).

### Data availability.

The raw data are in the NCBI database under the Sequence Read Archive accession numbers SRX4149050 (Coco) and SRX4150484 (NI). Quality-filtered and annotated data are in MG-RAST at mgm4815836.3 (Coco) and mgm4816434.3 (NI).
